# Improvement in mental and physical well-being among problematic substance users in Internet-based intervention trials

**DOI:** 10.1186/1940-0640-8-S1-A9

**Published:** 2013-09-04

**Authors:** Anne H Berman, Kristina Sinadinovic

**Affiliations:** 1 Karolinska Institute, Department of Clinical Neuroscience, Center for Psychiatric Research, Stockholm, Sweden; 2 Stockholm Center for Dependency Disorders, Stockholm, Sweden

## 

Changes in substance use should lead to subsequent improvements in health, but this topic is infrequently studied empirically. In this study, health effects were analyzed for a total of 835 Internet help-seekers who participated in one of two randomized controlled trials (RCTs) to reduce either alcohol or drug use following online assessment and brief intervention. Health effects over 12 months for participants who reduced their use to a clinically less problematic category were compared to health status for participants who maintained their use or increased it. Participants’ alcohol use was measured with the Alcohol Use Disorders Identification Test (AUDIT) and drug use was measured with the Drug Use Disorders identification Test (DUDIT). A battery of previously validated questions assessed experienced levels of general health. Health effects were analyzed using a repeated measures general linear model. The 633 participants with only problematic alcohol use (alcohol RCT), 55% women and 45% men, showed initial AUDIT scores suggesting alcohol dependence. The 202 problematic drug users (drug RCT), 45% women and 55% men, had initial AUDIT scores suggesting alcohol dependence and DUDIT scores suggesting harmful use of drugs. Participants from both trials showed low health outcome measures at baseline, with drug RCT participants generally lower in health compared to alcohol RCT participants. At the 12-month follow-up, about 35% of participants in both RCTs had reduced their substance use. In comparison to participants with unchanged or increase substance use, those with reduced alcohol use over 12 months reported positive health outcomes in wellbeing, sleep, concentration, energy, social life, sadness and current life meaning; those with reduced drug use reported better sleep, concentration and less sadness. Results were limited by significant attrition, typical to internet-based treatment studies. Our future studies will highlight both treatment- and health-related outcomes.

